# Cardiovascular Mechano-Epigenetics: Force-Dependent Regulation of Histone Modifications and Gene Regulation

**DOI:** 10.1007/s10557-022-07422-z

**Published:** 2023-01-18

**Authors:** Pamela Swiatlowska, Thomas Iskratsch

**Affiliations:** School of Engineering and Materials Science, Queen Mary University of London, London, UK

**Keywords:** Cardiovascular diseases, Mechanobiology, Mechano-epigenetics, Histone modifications

## Abstract

The local mechanical microenvironment impacts on the cell behavior. In the cardiovascular system, cells in both the heart and the vessels are exposed to continuous blood flow, blood pressure, stretching forces, and changing extracellular matrix stiffness. The force-induced signals travel all the way to the nucleus regulating epigenetic changes such as chromatin dynamics and gene expression. Mechanical cues are needed at the very early stage for a faultless embryological development, while later in life, aberrant mechanical signaling can lead to a range of pathologies, including diverse cardiovascular diseases. Hence, an investigation of force-generated epigenetic alteration at different time scales is needed to understand fully the phenotypic changes in disease onset and progression. That being so, cardiovascular mechano-epigenetics emerges as an attractive field of study. Given the rapid advances in this emergent field of research, this short review aims to provide an analysis of the state of knowledge of force-induced epigenetic changes in the cardiovascular field.

## Introduction

Cardiovascular diseases have become a worldwide epidemic and are a leading public health concern. Despite significant improvement in diagnostics and new treatments, the extensive complexity of cardiovascular pathologies drives the need for continuous effective drug discoveries.

Over the years, more light has been shed on the epigenetic mechanisms, highlighting the equal importance to the genetic field. Epigenomics is widely known as changes in the gene expression in response to the interaction with the environment, first reported in 1942 by Conrad Waddington as heritable chromosomal change without DNA sequence alterations [1]. The three types of epigenetic alterations include DNA methylation, histone modification, and non-coding RNA. DNA methylation acts by adding a methyl group to the cytosine in a CpG island (5-methylcytosine) of the DNA sequence, resulting in the gene suppression. Histones, that carry the main role of keep a tightly packed DNA, are targeted by PTMs thereby directly regulating transcription. Non-coding RNA (ncRNA) is a functional RNA that is not translated into a protein but can regulate transcription in different ways. Depending on the length, the ncRNA family is divided into two groups: short non-coding RNA (siRNA, miRNA, piRNA) and lncRNA. Receiving a lot of attention in recent years, ncRNA stands as a promising therapy strategy for cardiovascular diseases [2]. Studies to date have shown that the epigenetic changes are prevalent during normal development but are also present in a number of pathologies, such as cancer [3, 4], mental [5, 6], and autoimmune diseases [7] as well as cardiovascular diseases [2].

In parallel, the growing field of mechanobiology and its recognition as an integral part of the biological system increases year-to-year [8]. The role of mechanical cues in the development and function of the cardiovascular system is well-established [9, 10]. Mechanical signals are present as early as the start of the heart formation process to grow a faultless four-chambered organ. Later, force-generating cardiomyocytes synchronously contract to maintain the heartbeat and to transport gases and nutrients as well as waste all over the body. Cell–cell contacts and extracellular matrix communication make cardiac myocytes quick in responding to changes in the mechanical environment. Similarly, vessel remodeling is also sensitive to changes in the local mechanical environment. Removing hemodynamic force results in disrupted vessel development [11]. However, low substrate rigidity and hypertensive pressure result in vascular smooth muscle cells (vSMC) podosome formation, the hallmarks of atherosclerosis [12]. This shows that the cardiovascular system is mechano-dependent and is critical across the scales.

The rising body of evidence shows that changes in the mechanical microenvironment can travel all the way to the cell nucleus affecting chromatin landscape. Integrins, the transmembrane linkers, deliver information from the extracellular matrix to the cell cytoskeleton. A robust connection between the nucleus and the cytoskeleton is through a protein complex called LINC that is directly associated with outer and inner nuclear membrane. Further, inner membrane adaptor proteins couple to the chromatin and provide a link to the cell membrane [13]. The interplay between mechanical forces and epigenetic marks has been shown [14]; however, the knowledge in the field of mechano-epigenetics is still very limited. This review explores the current state of knowledge about the landscape of cardiovascular histone epigenetic changes linked to mechanobiology.

## Histone Modifications

Histones carry a wide variety of modifications. More widely studied chromatin structural support protein PTMs include acetylation, methylation, phosphorylation, and ubiquitylation. Less is known about the citrullination, crotonylation, SUMOylation, and GlcNAcylation. Previous studies have shown that the addition of methyl groups results in a more compact chromatin structure, effectively causing gene silencing [15]. On the other hand, acetylation relaxes chromatin structure and activates transcription [16].

**Fig. 1:**
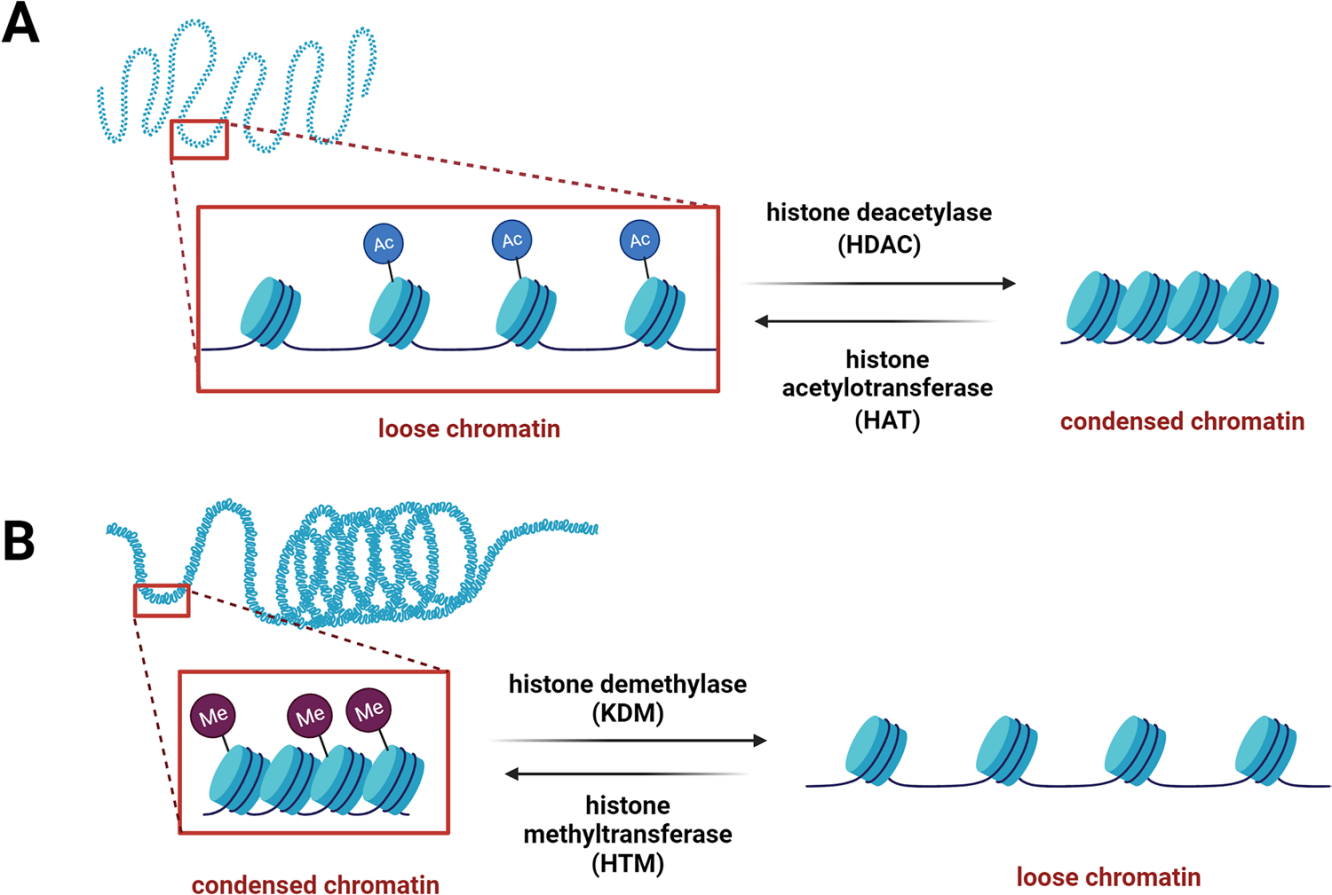
Enzyme families regulating acetylation (HDAC/HAT) and methylation (KDM/HTM) histones. **A** Histone acetylation by histone acetyltransferases leads to a decondensation of chromatin, while histone deacetylases (HDACs) remove acetylation resulting in a condensed state of the chromatin. **B** An opposite effect is seen for methylation of histones, catalyzed by histone methyltransferases (HTM), leading to a condensed chromatin, while demethylation by histone demethylases (KDM) leading to a loser chromatin. Created with Biorender.com

The addition and removal of the chemical groups are regulated through specific set of enzymes (Fig. 1).

## Chromatin Landscape in the Altered Mechanical Environment

The cross-talk between force and epigenetic marks has been identified in a number of different cell types. Altered mechanical properties of the microenvironment resulted in transcriptional repression of the adult hair follicle stem cells [17]. Keratinocyte–ECM adhesion on micropatterned surfaces regulates chromatin remodeling with less H3K27ac and bigger H3K9me3 foci on smaller surfaces [18]. Biaxial cyclic stretch reveals both global chromatin rearrangements and transcriptional repression by accumulation of H3K27me2 on lineage genes promoters, in human primary epidermal keratinocytes [19]. In the work from Nava et al., epidermis progenitor cell monolayer was subjected to uniaxial stretch at different amplitudes and time points. As quick as 30 min after 40% stretch biaxial application, H3k9me2 and − 3 signatures were decreased that recovered after 6 h. On the other hand, H3K27me3 showed no difference after one hour of stretch, but when subjected for a 24-h period of lower, 5 percent stretch, the values were significantly higher [14]. By labeling H2B with a GFP tag, Tajik and colleagues applied stress at 0 and 90 °C, using a 4.0-μm RGD-coated magnetic bead, bound to the surface of B16 melanoma cell line and CHO ovary cells. The study demonstrated that base level stress can cause chromatin stretching, further showing dihydrofolate reductase gene transcription upregulation with increased chromatin stretching [20]. On the other hand, squeezing cells through a 7-µm microchannel decreased the H3K9me histone mark. The effect persisted up to 12 h after stress application [21].

## Arterial Hemodynamics—Epigenetic Adaptability

Blood flow exerts hemodynamic force upon the arterial wall. The innermost layer, the vascular endothelium, is subjected to shear stress, a direct frictional force. On a linear route, the course of blood is continuous with a laminar flow pattern, but when it reaches a branched point or any curvatures, this can lead to a disturbed pattern further affecting the surrounding environment. Importantly, flow pattern has been shown to modulate the function of endothelial cells, defining atheroprone and atheroprotected regions of the arteries [22]. Hemodynamic forces also lead to distension of the arterial wall and pressure on the residing cells, which is experienced by vSMCs in the intima and media layers, together with changes in the extracellular matrix composition and stiffness [12]. Collectively, this shows that vascular biology is finely tuned to the mechanical stimuli acting on the cells and tissue.

Recent years of studies have shown a strong effect of hemodynamic forces on the histone epigenetic modifications in the endothelial cell (EC) biology. ECs that experienced atheroprotective, pulsative shear stress exhibited H3K27ac enrichment as analyzed by ChIP-seq, RNA-seq, and ATAC-seq. This marker of active chromatin state was specifically increased at the KLF4 promoter region, major regulator of EC homeostasis [23] with a further effect on endothelial nitric oxide synthase activation. Looking broadly at the epigenomic landscape, Andueza and colleagues demonstrated that disturbed flow reprograms EC in a mouse partial carotid ligation model. Changes were present at both, transcriptional and epigenetic level, leading to endothelial-to-mesenchymal transition, endothelial-to-immune-like cell transition, and endothelial-to-hematopoietic phenotypes [24].

A significant portion of histone mechano-epigenetic studies have focused on HDACs. The dynamics of the blood flow both, laminar and oscillatory, have an impact on the class 1 HDAC 1/2/3 activity that further govern the EC cycle, proliferation, and survival [25–28].

**Fig. 2:**
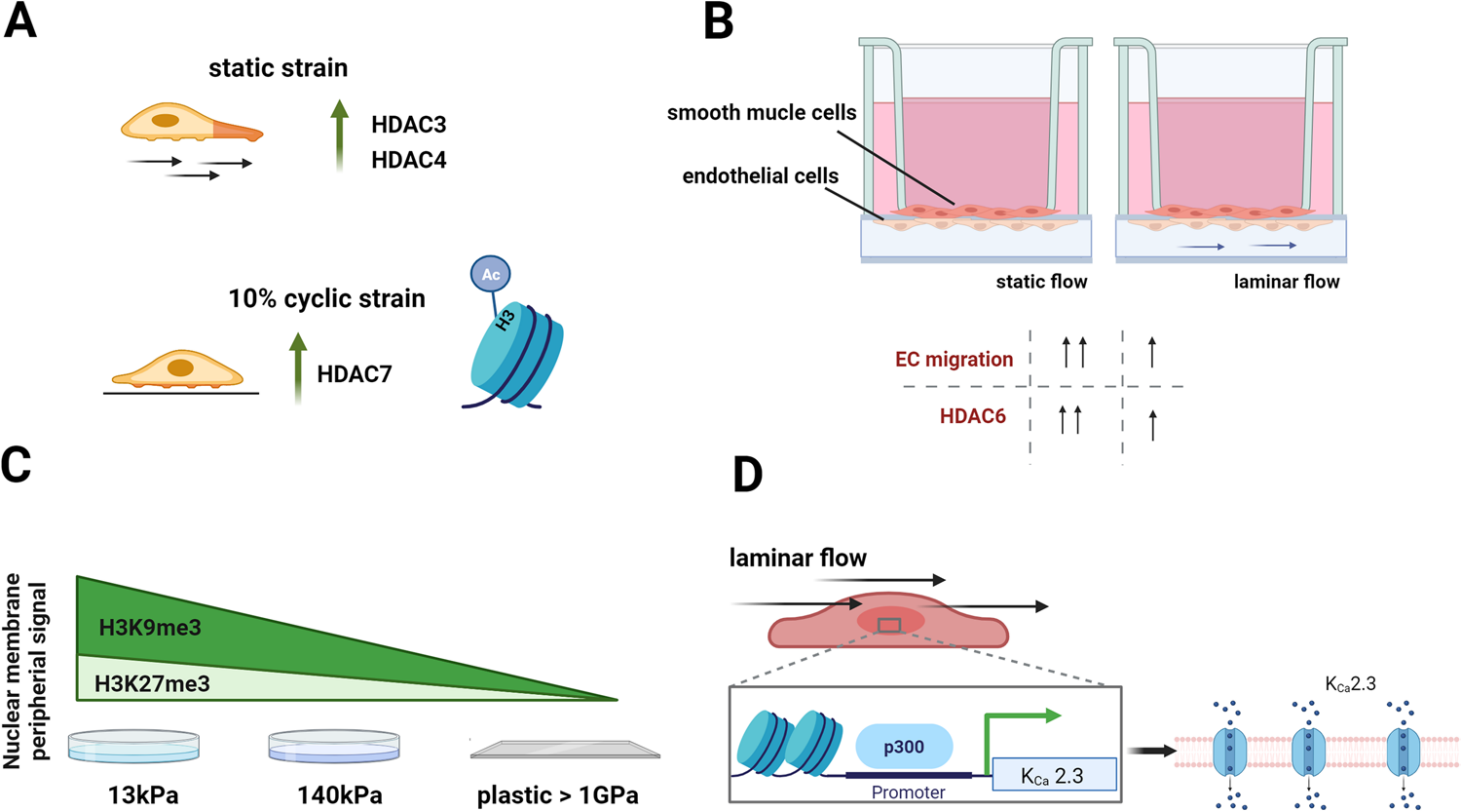
Force-dependent epigenetic changes. **A** Static strain-induced migration and higher HDAC3/4 levels in smooth muscle cells. **B** Static flow application to a smooth muscle cell–endothelial cell co-culture results in higher EC migration and higher HDAC6 level. **C** Embryonic cardiomyocytes cultured on 13-kPa PDMS show higher H3K9me3 peripheral nuclear marks. **D** Laminar flow increases *K*_Ca_ 2.3 level in H92c cells downstream of the p300 HAT. Created with Biorender.com

Similarly, class 2 HDACs are also prone to mechanical inputs. Application of 10% cyclic physiological stretch did not induce vSMC migration, an opposite effect that was observed under static strain (Fig. 2a). Lack of migratory properties came together with increased HDAC7 and H3 acetylation as well as decrease in HDAC3/4 [29]. Wang et al. looked at the EC-vSMC interaction in a parallel-plate co-culture flow chamber system (Fig. 2b). Under co-culture conditions, normal laminar flow application led to increased expression of HDAC6 and decreased acetylated tubulin in ECs. This effectively resulted in a reduction of EC migration when examined by a microporous membrane transwell migration assay [30]. In endothelial cells, flow-induced HDAC5 has been shown to improve KLF2 and eNOS expression, key atheroprotective players [31].

One of the most prominent members of class 3 HDACs is SIRT1, the NAD + -dependent deacetylase. Due to its role in a number of different diseases, such as ageing, cancer, neurodegeneration, and obesity, it attracted a considerable attention over the years [32]. Work from Kitada et al. reported that SIRT1, NAD^+^-dependent deacetylase can shelter EC, vSMC, and macrophages from various cell stressors, e.g., inflammation [33]. Twelve-hour laminar flow application increased level of SIRT1 that inhibits YAP acetylation, effectively facilitating nuclear export and autophagic degradation. On the other hand, 12-hour disturbed flow (DF) did not show any difference in SIRT1 level. However, the SIRT1 overexpression in the DF samples blocked YAP accumulation in the nucleus, thus exerting an atheroprotective effect [34]. Furthermore, histone acetylation through histone acetyltransferases (HAT) has also been reported to be dependent on force. Laminar shear stress activated the HAT p300 and increased acetylation of H3 and H4 histones in the eNOS promoter laminar shear stress response element, demonstrating an open chromatin structure [35].

Due to high plasticity capabilities, vSMC undergo phenotypic switching in a pathophysiological setting, such as atherosclerosis [36]. During this process, vSMC acquire proliferative and migratory properties as well as start to express marker genes characteristic to other cell types such as macrophage-, myofibroblast-, and chondro-/osteogenic-like cells [37–39]. Along with the genetic changes, substantial modifications in the epigenome are also present [40]. In the atherosclerotic setting, a downregulation of H3K9 and H3K27 methylation with increase in H3K9 and H3K27 acetylation is observed [40]. Moreover, recent work from Liu and colleagues introduced the H3K4me2 mark as a part of a vSMC lineage memory mechanism able to reverse temporary differentiation [41]. Overall, changes in the vSMC histone epigenetics are prevalent, and further investigation is needed to differentiate the force-dependent signature. A genome-wide study to investigate new genetic loci influencing blood pressure phenotypes in 320,251 participants indicated that the epigenetic regulator, PRDM6, a smooth muscle cell–specific histone methyltransferase, played a significant role, through regulating vSMC function [42].

## Force-Dependent Epigenetic Regulation in the Heart

Within the cardiac tissue, multiple studies have provided evidence of histone methylation and demethylation in cardiac pathologies [43]. However, which epigenetic marks are modified by force is largely unknown.

Recent work from Finke et al. show that H3K9 methylation was associated with exercise-induced gene expression in cardiac myocytes [44]. Further, JMJD2A, the H3K9me3 demethylase, was reported to promote cardiac hypertrophy in response to hypertrophic stimuli and activate transcription of genes characteristic to hypertrophic pathology [45].

In an in vitro cardiomyocyte maturation model, where embryonic cardiomyocytes were plated on soft (13 kPa) or stiff (140 kPa) PDMS, Seelbinder and co-workers showed that intranuclear tension spatially correlated with the H3K9me3 chromatin mark (Fig. 2c). They further demonstrated that suppression of H3K9 methylation induced chromatin rearrangement and decreased the expression of cardiac developmental gene [46]. Zhang et al., studying HDAC knock out mice, showed that class II HDACs, specifically HDAC9, are sensitive to stress signals, as the HDAC9 knockout mouse model exhibited an excessive response to hypertrophic stimuli. However, mutation of HDAC9 conserved CaMK phosphorylation sites turned cardiomyocytes to being resistant to hypertrophic response when subjected to phenylephrine [47]. In atrial fibrillation patients, Li et al. demonstrated an upregulation in Ca^2+^-activated K^+^ channels (Fig. 2d). This increase was linked to laminar shear stress-activated p300 HAT [48].

## Tools for Mechano-Epigenetics

With an increased interest in mechanobiology, there is a wide range of techniques available to probe mechanical properties, addressing different ranges from the nanoscale to microscale and macroscale [12, 49]. In our previous work [49], we took a close look at these methods and examined carefully their applications. Our review on mechanobiology tools guides the reader through different techniques and helps to choose the right method in order to answer the research question. Access to such a variety of technologies addressing cross scale mechanical responses, provides us with a comprehensive understanding of the cardiac mechanobiology.

The concept of mechanosensation in the cardiovascular system has become progressively more coherent. To make even further progress, these tools should be now combined with other assays to study force-dependent changes.

The blooming field of epigenetics evolved over the years along with its different experimental techniques to better understand the mechanism of phenotype change without altering the DNA sequence. Apart from conventional immunolabeling, western blot, and ELISA using specific histone PTMs antibodies, more in-depth techniques have been designed.

The standard technique to analyze DNA–protein interactions, where transcription factor, histones, and histone modifications are selectively recognized, is ChIP. Following the cross-linking and cleavage steps, antibody-bead constructs are added and centrifuged to further separate the 200–800 base pair DNA sequences. Purified DNA is then used for qPCR, sequencing, or microarray to determine the sequence. However, the high number of cells and a separate library preparation is required, making it a more challenging technique. ChIP-chip is a sister technique that is also based on chromatin immunoprecipitation but requires a microarray for hybridization. Due to its limitations regarding resolution, amount of sample, lower dynamic range, and overall array scaled-down sample, it was only rarely used and has been now replaced with newer methods [50].

For epigenomic make-up investigation and looking at chromatin accessibility with loose nucleosome regions, ATAC-sequencing is commonly used. Tn5 transposase binds to accessible DNA that is further fragmented, amplified, and sequenced.

In 2019, Kaya-Okur et al. presented a new tool to map chromatin called “CUT&Tag” (Cleavage Under Targets and Tagmentation), an improved version of “CUT&Run” (Cleavage Under Targets & Release Using Nuclease) [51, 52]. It is based on nuclear extraction and immobilization on a solid support where histone PTMs are labeled with specific antibodies. During the following tagmentation, a fusion protein of protein A, protein G, and Tn5 transposase binds to the antibodies and catalyzes simultaneously the fragmentation of the DNA and the attachment of sequencing adapters in each cut. In contrast, in CUT&Run, cells are immobilized on magnetic beads, permeabilized, and treated with calcium-activated protein A/G micrococcal nuclease which then cuts the DNA. With a better resolution compared to its predecessors, CUT&Tag requires less working material, and the in situ*-*built library reduces the time of the experiment to 1–2 days. It is well suited for histone marks analysis, but the required high-salt buffers are not ideal for investigating transcription factors.

Epigenetic profiling is also possible thanks to recently introduced high-throughput mass spectrometry-based technique that finds all histone PTMs in a label-free approach [53].

Now, with more and more live-cell imaging, probes monitoring chromatin changes became easier [54]. Recent years have shown FRET-based biosensors as an effective tool in studying mechanobiology by tracking the mechanotransduction in live cells [43]. These fluorescent reporters have also found its application in tracking epigenetic changes. So far, a number of different energy-transfer probes have been reported that target different histone PTMs, such as methylation, acetylation, and phosphorylation [55–63]. High specificity and sensitivity with elevated temporal and spatial resolution make this tool an easy approach to investigate epigenetic modifications in real-time.

## Therapeutical Approach

Owing to the reversible manner, epigenetic marks offer a great therapeutic potential. Using epigenetics as biomarkers has been growing for years now in diabetes [64], osteoarthritis [65], cancer [66], and cardiovascular diseases [67]. Epigenetic-targeted therapies are inviting a lot of attention as an anticancer strategy. In mixed lineage leukemia therapy, H3K79 methyltransferase that regulates gene expression was targeted by a small molecule inhibitor, EPC-5676, effectively resulting in lower histone H3 lysine 79 methylation and mixed-lineage leukemia gene expression level [68]. Some HDACi have been approved as cancer treatment. However, the side effects and toxicity are under close investigation of these drugs [69, 70]. Nevertheless, a combination therapy, HDACi with other therapeutic approaches or agents, shows a new more promising path.

In the cardiovascular field, epidrugs have been applied in different pathologies. H3K27me3, marker of the gene repression, has been observed in the distal artery wall of hypertensive rats. Resveratrol treatment resulted in increased H3K27me3 signal across all the aorta layers [71]. On the other hand, C646 molecule has shown a potential to inhibit HAT p300, subsequently blocking the calcification of aortic valves [72]. Valproic acid, class I and to a lesser extent class II HDACi, reduced the infarct size by 50% in acute heart failure and improved the heart performance in longer term [73]. Also, alleviated fibrotic and proinflammatory responses were observed in heart failure [74].

The implication of ncRNA in CVD diseases and emerging as potential therapeutic target has taken the stage in the last years [2, 75, 76]. All in all, lot of progress has been made in the field, leading to several candidates being examined in clinical trials, targeting DNA methylation, histone modifications, and ncRNA. Inhibition of miRNA-92a (MRG-110 inhibitor) has shown in phase I clinical trials increase in number and size of blood vessels, demonstrating a pro-angiogenic effect [77]. Clinical data also show that acetylsalicylic acid therapy lowers DNA methylation of ABCA1 gene responsible for lipid homeostasis critical in the cardiovascular diseases [78]. Very thorough reviews from Shi et al. and Laggerbauer and Engelhardt have been published recently, summarizing the recent advances in targeting epigenetic changes in cardiovascular diseases [2, 76].

It is important to note that cardiovascular epidrugs are not limited to histone modification inhibitors. RG108, a selective small molecule inhibitor, binds to DNA methyltransferase1 and 3a, a potential target in coronary heart disease and atherosclerosis [70]. Also hydralazine, primary used as a vasodilator, has been repurposed as epidrug, due to its ability to cause DNA hypomethylation [79]. The repurposing is an attractive approach as a fast-track, lower-cost path with already available safety data and pharmacokinetic. However, this approach comes with its own challenges involving closer industrial collaboration to access clinical trial libraries, new platforms, or new funding schemes [80].

Despite a big potential as a therapeutic target, epigenetic therapies still carry some difficulties. One of the main obstacles that the field is facing is the broad spectrum of action, affecting different cell types and organs. Therefore, future medical treatments will require addressing cell-specificity as well as side effects.

## Conclusions

Understanding the force-driven epigenetic changes will provide us with fundamental information on gene expression. Incorporating mechanobiology tools and techniques for probing epigenomics will be the right step in that direction by further building up the field of mechano-epigenetics.

Changes in the nucleosomes are rapid and can differ at various time points [14] as well as depend on the mechanical stimuli [14, 20, 81]. Miroshnikova and colleagues present chromatin as a viscoelastic rheological model that can easily adapt when subjected to biomechanical cues. Short-term effects allow to adjust to the changing microenvironment (open state), but when the effect persists, gene silencing takes off (closed state) [82].

In recent years, miRNAs have been widely recognized as potential CVD therapeutics. Therefore, looking at the entire spectrum of force-induced epigenetic changes, especially histone acetylases, histone deacetylases, DNA methyltransferases, and histone methyltransferases, holds a lot of promise for future cardiovascular research and therapeutics.

Owing to the fact that cardiovascular system is prone to mechanical stimulation, mechano-epigenetics is an attractive area of research that needs further investigation in health and disease.

## Data Availability

Not applicable

## Abbreviations

ABCA1: ATP-binding cassette transporter A1
ATAC-seq: Assay for transposase-accessible chromatin sequencing
ChIP-chip: Chromatin immunoprecipitation on an microarray
ChIP-seq: Chromatin immunoprecipitation sequencing
CpG island: 200 base pair region of DNA with a GC content higher than 50%C
CUT&Tag: Cleavage Under Targets and Tagmentation
CUT&Run: Cleavage Under Targets & Release Using Nuclease
EC: Endothelial cells
H3K27ac: Histone 3 lysine 27 acetylation
H3K27me2: Histone 3 lysine 9 di-methylation
H3K9me3: Histone 3 lysine 9 tri-methylation
H3K27me3: Histone 3 lysine 27 tri-methylation
HDAC: Histone deacetylase
HDACi: HDAC inhibitors
HAT: Histone acetyltransferase
LINC: Linker of Nucleoskeleton and Cytoskeleton
miRNA: Micro-RNA
ncRNA: Non-coding RNA
PDMS: Polydimethylsiloxane
piRNA: Piwi-interacting RNA
PTMs: Posttranslational modifications
siRNA: Small-interfering RNA
